# Antiphospholipid antibodies in patients with dysglycaemia: A neglected cardiovascular risk factor?

**DOI:** 10.1177/1479164120922123

**Published:** 2020-06-07

**Authors:** Giulia Ferrannini, Elisabet Svenungsson, Barbro Kjellström, Kerstin Elvin, Giorgia Grosso, Per Näsman, Lars Rydén, Anna Norhammar

**Affiliations:** 1 Cardiology Unit, Department of Medicine Solna, Karolinska Institutet, Karolinska University Hospital, Stockholm, Sweden; 2 Rheumatology Unit, Department of Medicine Solna, Karolinska Institutet, Karolinska University Hospital, Stockholm, Sweden; 3 Division of Immunology and Allergy, Department of Medicine Solna, Karolinska Institutet, Karolinska University Hospital, Stockholm, Sweden; 4 Centre for Safety Research, KTH Royal Institute of Technology, Stockholm, Sweden; 5 Capio Saint Görans Hospital, Stockholm, Sweden

**Keywords:** Antiphospholipid antibodies, antithrombotic agents, diabetes mellitus, myocardial infarction, glucose tolerance test

## Abstract

**Background:**

Cardiovascular disease is a serious complication in patients with dysglycaemia, defined as either type 2 diabetes or impaired glucose tolerance. Research focusing on the identification of potential markers for atherothrombotic disease in these subjects is warranted. The antiphospholipid syndrome is a common acquired prothrombotic condition, defined by a combination of thrombotic events and/or obstetric morbidity and positivity of specific antiphospholipid antibodies. Available information on antiphospholipid antibodies in dysglycaemia is scarce.

**Objective:**

This study investigates the association between antiphospholipid antibodies and dysglycaemia.

**Patients/Methods:**

The PAROKRANK (periodontitis and its relation to coronary artery disease) study included 805 patients, investigated 6–10 weeks after a first myocardial infarction, and 805 matched controls. Participants without known diabetes (91%) underwent an oral glucose tolerance test. Associations between antiphospholipid antibodies (anti-cardiolipin and anti-β2 glycoprotein-I IgG, IgM and IgA) and dysglycaemia were analysed.

**Results:**

In total, 137 (9%) subjects had previously known type 2 diabetes and 371 (23%) newly diagnosed dysglycaemia. Compared with the normoglycaemic participants, those with dysglycaemia had a higher proportion with first myocardial infarction (61% vs 45%, *p* < 0.0001) and were more often antiphospholipid antibody IgG positive (8% vs 5%; *p* = 0.013). HbA1c, fasting glucose and 2-h glucose were significantly associated to antiphospholipid antibody IgG. Odds ratios (ORs) were 1.04 (95% confidence interval [CI] 1.02–1.06), 1.14 (95% CI 1.00 – 1.27) and 1.12 (95% CI 1.04 – 1.21), respectively, after adjustments for age, gender and smoking.

**Conclusions:**

This study reports an association between antiphospholipid antibody IgG positivity and dysglycaemia. Further studies are needed to verify these findings and to investigate if antithrombotic therapy reduces vascular complications in antiphospholipid antibody positive subjects with dysglycaemia.

## Background

Cardiovascular disease (CVD) is the leading global mortality cause, with the vast majority of deaths being related to the atherosclerotic vascular disease.^1,2^ People with dysglycaemia, defined as either type 2 diabetes or impaired glucose tolerance (IGT), are at a two to four times higher risk for cardiovascular events compared with the general population and CVD accounts for about 50% of all mortality in this patient group.^3,4^ This enhanced risk is primarily explained by the ‘common soil’ shared by CVD and dysglycaemia, which describes the clustering of vascular risk factors (endothelial dysfunction, increased platelet activity, suppression of fibrinolytic capacity, hyperglycaemia, dyslipidaemia and hypertension) around insulin resistance.^5,6^ Nevertheless, prediction models based on traditional risk factors fail to stratify their CVD risk even if recent studies have improved precision medicine in this field.^7,8^ In this framework, the assessment of thrombophilic factors might help to further characterise the CVD risk in the heterogeneous diabetes population.

Among thrombophilic conditions, the antiphospholipid syndrome (APS) is one of the most common. APS is defined by positivity in specific tests for antiphospholipid antibodies (aPL) together with clinical manifestation of arterial and/or venous and/or small vessel thromboses and/or obstetric morbidity.^9^ The Sidney criteria for classification of APS include the three ‘classic’ aPL tests: the functional lupus anticoagulant (LA), and specific tests for anti-β_2_-glycoprotein I (anti-β_2_GPI, IgG/M) and anti-cardiolipin (aCL, IgG/M) antibodies.^10^ Positivity for any of these tests needs to be confirmed at least twice with a minimum interval of 12 weeks.^10^ In addition, several other ‘non-criteria aPL’, for example, anti-phosphatidylserine/prothrombin, anti-phosphatidylethanolamine, anti-phosphatidylinositol and anti-phosphatidylcholine are studied and may have a role in the syndrome.^10^ In the last 15 years, considerable effort has been put into developing international standards for such aPL testing to improve diagnostic accuracy of APS patients.^9^ Thanks to these advancements, the 2019 European League Against Rheumatism (EULAR) guidelines for the management of APS identified ‘high-risk profiles’, taking into account the aPL type, the presence of positivity in multiple versus single aPL tests, high versus low titres and the persistence of their positivity in repeated measurements.^11^ This is of pivotal importance, since high-risk profiles require more intensive treatment, both for primary and secondary prevention of vascular events.^11^ In addition, the EULAR guidelines strongly recommend screening for traditional cardiovascular risk factors, including diabetes, in aPL positive patients, because their coexistence is considered to increase the risk of vascular events.^11^

However, the information on the prevalence of aPL both in the general population and in patients with dysglycaemia, especially if previously undetected, is still sparse, and their association with vascular complications are, at least partially, outdated and conflicting.^12–18^ The assessment of aPL in dysglycaemic conditions is of interest, because they activate pathophysiological pathways leading to increased systemic inflammation and thrombophilia, making their possible involvement as independent markers of cardiovascular risk worthwhile.^18^

The objective of this investigation, based on the PAROKRANK (periodontitis and its relation to coronary artery disease) population,^19^ is to test the hypothesis that there is an association between aPL and dysglycaemia, including both known and unknown glucose perturbations. If this is true, aPL testing may identify a subgroup of patients with dysglycaemia, who may benefit from antithrombotic treatment.

## Methods

### Study population

PAROKRANK, a multicentre case–control study, enrolled 1610 participants from 17 Swedish hospitals from May 2010 to February 2014.^19^ A total of 805 patients ⩽75 years old and with a first-time myocardial infarction according to international criteria were recruited, following informed consent.^19^ Exclusion criteria were prior myocardial infarction, heart valve replacement and any other condition that might limit the ability to adhere to the study protocol.

The control subjects (n = 805) were randomly selected from the national population registry and individually matched to patients for age (±3 months), gender and postal code area. They had to be free from previous myocardial infarction and heart valve replacement and willing to participate.

The study was coordinated from the Cardiology Unit, Department of Medicine at Karolinska Institutet, Stockholm, Sweden. A detailed delineation of the PAROKRANK study has been published elsewhere^19^ and this description is focused on features related to the present report.

### Study protocol

Patients were recruited during or in close connection to the hospitalisation for the myocardial infarction and were scheduled for an outpatient visits 6 to 10 weeks later at the local department of cardiology. To complete the investigations during the same season, the matched control subjects were selected and investigated soon after the outpatient visit of their corresponding patients. All participants fasted and abstained from smoking for 12 h before blood samples were collected and a physical examination was performed. Questionnaires comprising extensive information on family and medical history, risk and health preserving factors were completed. Smoking habits were defined as current, previous (stopped > 1 month ago) or never.

### Laboratory analyses

Blood samples were collected during the study visit 6 to 10 weeks after the myocardial infarction in patients and at baseline in controls. The following analyses were performed at the local laboratory: complete blood count, triglycerides, fibrinogen, glucose and HbA1c. High sensitivity C-reactive protein (hsCRP) was analysed at a central laboratory (Redhot Diagnostics, Södertälje, Sweden) with an enzyme-linked immunosorbent assay method (ELISA; MP Biomedicals, New York, USA) intended for quantitative determination C-reactive protein, with the functional sensitivity of 0.1 mg/L. Plasma were stored at −70°C in a central biobank at Karolinska Institutet.

#### Antiphospholipid antibodies

Antiphospholipid antibodies, including anti-cardiolipin and anti-β_2_-glycoprotein1 (IgG, IgM, IgA), were analysed from stored plasma by multiplexed bead technology (Luminex) using BioPlex 2200 system (Bio-Rad, Hercules, CA, USA) according to the specifications of the manufacturer. Study participants with self-reported type 1 diabetes (n = 5) were excluded.

The coefficient of variation % was < 8.0 E/mL for all isotypes. The cut-off for anti-CL and anti-β_2_GPI positivity was set at the 99th percentile of the normal population, according to APS criteria.^10^

Antibodies to specific nuclear antigens, that is., antinuclear antibodies (including dsDNA, nucleosomes, Smith antigen, Smith antigen ribonucleoprotein, ribosomal P protein, ribonucleoproteins 68 and A, Sjögren-syndrome antigen A Ro-52 and Ro-60, Sjögren’ syndrome antigen B) were also analysed by multiplexed bead technology (Luminex) using BioPlex 2200 system (Bio-Rad, Hercules, CA, USA), in accordance with the specifications of the manufacturer.

### Definitions

#### aPL IgG positivity

The distribution of aPL in the PAROKRANK population demonstrated a strong correlation between aCL and aβ_2_GPI antibodies for each aPL isotype, IgG (r_s_ = 0.85), IgM (r_s_ = 0.92) and IgA (r_s_ = 0.86). Only aCL and aβ_2_GPI of the IgG isotype was associated with myocardial infarction (MI).^20^

In the present study, we therefore focused on aPL IgG positivity, defined as positivity for either IgG aCL and/or IgG aβ_2_GPI.

#### Glycaemic state

Study participants with self-reported type 1 diabetes (total n = 5) were excluded. Participants without previously known diabetes underwent a standardised oral glucose tolerance test (OGTT) consisting of 75 g glucose diluted in 200 ml of water.^19^ Venous plasma glucose was measured before ingestion of the glucose solution and 120 min after, using a bedside point of care system (HemoCue^®^ 201 System, HemoCue^®^ AB, Ängelholm, Sweden). Glucose levels obtained during the OGTT were used to classify study participants’ glycaemic state according to the World Health Organization (WHO)^21^ as outlined in Table 1.

**Table 1.** Definition of glycaemic state.

	Venous plasma glucose (mmol/L)	
	Fasting	2-h post-load
Normoglycaemic	<7.0	<7.8
Impaired glucose tolerance	<7.0	7.8–11.0
Diabetes	⩾7.0	>11.0
Dysglycaemia (impaired glucose tolerance + diabetes)	⩾7.0	⩾7.8

*Dysglycaemia* was defined as the presence of either previously known type 2 diabetes or newly detected Impaired Glucose Tolerance (IGT) or type 2 diabetes.

### Ethical approval

The study was approved by the Regional Ethics Committee at Stockholm (Dnr:2008/152-31/2) prior to the study. All study participants provided written informed consent to participate.

Patients were not invited to comment on the study design and were not consulted to develop relevant outcomes or interpret the results. Patients were not invited to contribute to the writing or editing of this document.

### Statistical methods and data analysis

Descriptive statistics were employed to characterise patients and controls. Continuous variables with a normal distribution were compared using the Student’s t-test for independent samples, whereas variables with a skewed distribution were compared by Mann–Whitney U tests for independent samples. Differences between groups were investigated the chi-square test in the case of nominal data. When expected frequencies were low, Fisher’s exact test was used. Odds ratios, crude and adjusted for confounders known to be associated with either diabetes or aPL (i.e. age, gender and smoking) and corresponding 95% confidence intervals were calculated by use of logistic regression to assess the association between glucose variables and aPL IgG positivity in the total cohort. Correlations were calculated using the Spearman rank correlation coefficient. Calculations were performed using SAS software (SAS system for Windows 9.4, SAS Institute Inc., Cary, NC, USA).

A two-sided *p*-value < 0.05 was considered statistically significant.

## Results

**Figure 1.**
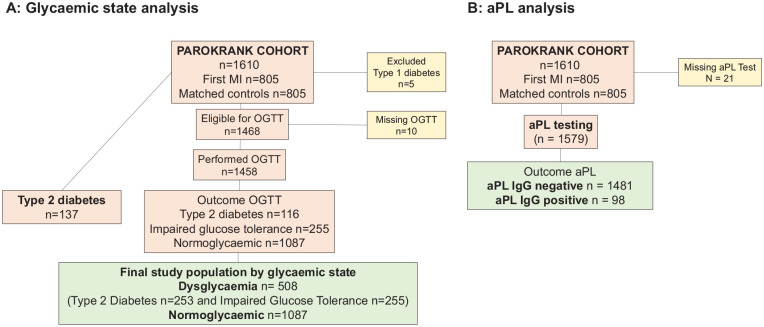
Flowchart for the analysis of study population by glycaemic state (panel A) and antiphospholipid antibodies testing (panel B). aPL: antiphospholipid antibodies; IgG: immunoglobulin G; MI: myocardial infarction; OGTT: Oral Glucose Tolerance Test; PAROKRANK: periodontitis and its relation to coronary artery disease.

A total of 137 subjects had previously known type 2 diabetes. When an OGTT was performed on the 1458 participants without such case history newly detected dysglycaemia was detected in 371 of them (25%) of whom 255 (69%) with IGT and 116 (31%) with type 2 diabetes. Thus, a total of 508 (35%) subjects were identified with dysglycaemia (Figure 1). Pertinent characteristics of the study population by glycaemic state are presented in Table 2. Dysglycaemic participants were older (mean age 64 ± 7 vs 61 ± 8 years; *p* < 0.001) and presented with a higher proportion of hypertension, rheumatic disease, pulmonary disease, history of cancer overweight, dyslipidaemia and elevated inflammatory markers (fibrinogen, white blood cell count and hsCRP) than the normoglycaemic group. Moreover, a higher proportion of participants with dysglycaemia had an index myocardial infarction than those with normoglycaemia (61% vs 45%, *p* < 0.0001).

**Table 2.** Baseline characteristics and proportion of aPL by glycaemic state in study participants.

Variables	Normoglycaemia n = 1087	Dysglycaemia^a^ n = 508	*p*-value
Age (years)	61 ± 8	64 ± 7	<0.001
Female gender	204 (19)	96 (19)	0.95
Index myocardial infarction	486 (45)	312 (61)	<0.0001
Family history of cardiovascular disease	335 (35)	147 (29)	0.28
Medical history			
Hypertension	283 (26)	264 (52)	<0.0001
Peripheral artery disease	15 (1)	9 (1)	0.52
Stroke	21 (2)	18 (4)	0.06
Rheumatic disease	184 (17)	113 (22)	0.015
Pulmonary disease	114 (11)	74 (15)	0.021
Kidney disease	37 (3)	26 (5)	0.13
Cancer	72 (7)	49 (10)	0.037
Depression	100 (9)	31 (8)	0.67
DVT and/or pulmonary embolism	36 (3)	22 (4)	0.32
Smoking habits (patients at admission)			
Current	206 (19)	85 (17)	
Previous	524 (48)	275 (54)	0.07
Never	357 (33)	146 (29)	
Waist circumference (cm)	97 ± 11	102 ± 12	<0.0001
Body mass index (kg/m^2^)	26 ± 4	28 ± 4	<0.0001
Laboratory			
Triglycerides (mmol/L)	1.3 ± 0.9	1.5 ± 1.3	0.0002
Fibrinogen (g/L)	3.2 ± 0.7	3.4 ± 0.9	<0.0001
High sensitivity CRP (mg/L)	2.0 ± 2.3	2.7 ± 3.1	<0.0001
White blood cell count (×10^9^/L)	5.9 ± 3.3	6.8 ± 5.2	<0.0001
Platelet count (×10^9^/L)	235 ± 57	240 ± 68	0.08
HbA1c [IFCC mmol/mol; (DCCT %)]	37 ± 4; (5.6 ± 2.5)	45 ± 11; (6.3 ± 3.2)	<0.0001
Glucose status			
Fasting plasma glucose (mmol/L)	5.4 ± 0.6	6.8 ± 1.8	<0.0001
OGTT 30’ (mmol/L)	8.6 ± 1.5	10.2 ± 2.0	<0.0001
OGTT 120’ (mmol/L)	5.5 ± 1.2	9.7 ± 2.3	<0.0001
Pharmacological treatment			
Renin-angiotensin inhibitors	519 (48)	370 (73)	<0.0001
Aspirin	508 (47)	341 (67)	<0.0001
Beta-blockers	492 (45)	340 (67)	<0.0001
Statins	535 (49)	361 (71)	< 0.0001
Anti-inflammatory agents	28 (3)	16 (3)	0.50
Corticosteroids	33 (3)	21 (4)	0.26
Antidepressants	70 (6)	23 (5)	0.12
Education			
1–12 years	668 (62)	347 (69)	0.008
University	414 (38)	159 (31)	
Antiphospholipid antibodies			
IgG aβ_2_GPI positivity	50 (5)	39 (8)	0.013
IgG aCL positivity	51 (5)	41 (8)	0.008
IgG aPL positivity (aβ_2_GPI and/or aCL)	55 (5)	42 (8)	0.013
IgA aβ_2_GPI positivity	12 (1)	9 (2)	0.29
IgA aCL positivity	12 (1)	9 (2)	0.29
IgM aβ_2_GPI positivity	12 (1)	6 (1)	0.89
IgM aCL positivity	12 (1)	8 (2)	0.44
IgG aCL titres	5.6 ± 21.2	8.0 ± 27.0	0.05
IgA aCL titres	2.3 ± 10.5	2.7 ± 11.4	0.52
IgM aCL titres	3.6 ± 10.7	3.5 ± 7.9	0.79
IgG aβ_2_GPI titres	5.1 ± 20.7	7.3 ± 26.1	0.07
IgA aβ_2_GPI titres	2.1 ± 9.7	2.4 ± 9.6	0.64
IgM aβ_2_GPI titres	3.4 ± 10.2	3.3 ± 7.3	0.91

aβ_2_GPI: anti-beta2-glycoprotein I antibodies; aCL: anti-cardiolipin antibodies; aPL: antiphospholipid antibodies; CRP: C-reactive protein; DCCT: Diabetes Control and Complications Trial standardisation of HbA1c; DVT: deep venous thrombosis; IFCC: International Federation of Clinical Chemistry standardisation of HbA1c; IgA: immunoglobulin A; IgG: immunoglobulin G; IgM: immunoglobulin M; OGTT: Oral Glucose Tolerance Test.

Data are presented as mean ± SD or number (%). If not otherwise stated, patient data were retrieved at the follow-up visit.

a Dysglycaemia includes known type 2 diabetes and newly detected IGT or diabetes on OGTT.

### aPL IgG positivity

IgG aCL or IgG aβ_2_GPI was assessed in 1579 of 1600 participants (missing: 21) (Figure 1). aPL IgG positivity was more common in the dysglycaemic group (8% vs 5%; *p* = 0.013, Table 2). Table 3 depicts baseline characteristics, medication use and glycaemic variables of participants with (n = 98) and without (n = 1481) aPL IgG positivity. The proportion of myocardial infarction was higher in the aPL IgG positive compared with the aPL IgG negative group (90% vs 47%; *p* < 0.001) as was the proportions of pulmonary disease and an increased white blood cell count. There was no difference between the two groups with regard to previous deep venous thrombosis, pulmonary embolism and rheumatic disease. A higher proportion of aPL IgG positive subjects was positive to at least one antinuclear antigen as compared with the aPL IgG negative group (16% vs 10%, *p* = 0.047, missing = 3). Furthermore, dysglycaemia was more common in the aPL IgG positive group (43% vs 31%; *p* = 0.018). HbA1c and fasting glucose were assessed in the whole cohort, whereas 2-h post-load glucose levels were measured only in subjects without previously known diabetes. HbA1c, fasting glucose levels and 2-h post-load glucose levels were significantly higher in the aPL IgG positive group compared with the aPL IgG negative group.

**Table 3.** Clinical characteristics of the study population by antiphospholipid antibody (aPL) IgG positivity.

Variables	aPL IgG positive n = 98	aPL IgG negative n = 1481	*p*-value
Age (years)	63 ± 6	62 ± 8	0.22
Female gender	23 (23)	275 (19)	0.23
Index myocardial infarction	88 (90)	703 (47)	<0.001
Family history of cardiovascular disease	30 (31)	445 (30)	0.6
Dysglycaemia	42 (43)	457 (31)	0.018
Medical history			
Hypertension	27 (28)	515 (35)	0.15
Peripheral artery disease	2 (2)	26 (2)	0.83
Stroke	2 (2)	37 (3)	0.75
Diabetes mellitus	11 (11)	124 (8)	0.32
Rheumatic disease	23 (24)	273 (19)	0.19
Pulmonary disease	22 (23)	167 (11)	<0.0001
Kidney disease	6 (6)	56 (4)	0.24
Cancer	9 (9)	111 (7)	0.5
Depression	8 (8)	134 (9)	0.76
DVT and/or pulmonary embolism	2 (2)	56 (4)	0.38
Smoking habits			
Current	20 (20)	271 (18)	
Previous	52 (53)	735 (50)	0.5
Never	26 (26)	473 (32)	
Waist circumference (cm)	99 ± 14	99 ± 11	0.64
Body Mass Index (kg/m^2^)	27 ± 5	27 ± 4	0.33
Laboratory			
Triglycerides (mmol/L)	1.2 ± 0.7	1.4 ± 1.1	0.18
Fibrinogen (g/L)	3.4 ± 0.9	3.3 ± 0.8	0.08
High sensitivity CRP (mg/L)	2.4 ± 2.6	2.2 ± 2.6	0.49
White blood cell count (×10^9^/L)	7.6 ± 10.4	6.1 ± 3.2	<0.0001
Platelet count (×10^9^/L)	231 ± 50	237 ± 62	0.39
HbA1c [IFCC mmol/mol; (DCCT %)]	43 ± 10 (6.1 ± 3.1)	40 ± 8 (5.9 ± 2.9)	<0.0001
IgG aCL titres	56.0 [22.7–138.8]	1.5 [1.5–1.5]	<0.0001
IgA aCL titres	1.4 [0.6–4.5]	0.7 [0.4–1.4]	<0.0001
IgM aCL titres	1.8 [0.6–4.4]	1.1 [0.4–3.1]	<0.0001
IgG aβ_2_GPI titres	49.6 [19.8–132.5]	1.3 [1.3–1.3]	<0.0001
IgA aβ_2_GPI titres	1.1 [0.6–4.3]	0.6 [0.5–1.2]	<0.0001
IgM aβ_2_GPI titres	1.8 [0.6–5.2]	1.1 [0.4–2.9]	<0.0001
Glucose status			
Fasting plasma glucose (mmol/L)	6.1 ± 1.4	5.8 ± 1.3	0.020
OGTT 30’ (mmol/L)	9.7 ± 1.9	8.9 ± 1.8	<0.0001
OGTT 120’ (mmol/L)	7.4 ± 3.1	6.5 ± 2.4	0.0018
Pharmacological treatment			
Renin-angiotensin inhibitors	77 (79)	802 (54)	<0.001
Aspirin	85 (87)	756 (51)	<0.001
Beta-blockers	78 (80)	746 (50)	<0.001
Statins	83 (85)	805 (54)	<0.001
Anti-inflammatory agents	1 (1)	43 (3)	0.26
Corticosteroids	3 (3)	51 (3)	0.87
Antidepressants	4 (4)	89 (6)	0.46
Glucose tolerance status			
Newly detected IGT	18 (18)	233 (16)	0.45
Newly detected diabetes	13 (13)	100 (7)	0.013
Newly detected dysglycaemia	31 (32)	333 (22)	0.03
Autoantibodies targeting specific nuclear antigens (ANA)			
dsDNA	3 (3)	30 (2)	0.49
Nucleosomes	1 (1)	7 (0.5)	0.46
Sm	0 (0)	1 (0.1)	N.A.
SmRNP	1 (1)	3 (0.2)	0.12
Ribosomal P	0 (0)	0 (0)	N.A.
RNP 68	0 (0)	4 (0.3)	N.A.
RNP A	2 (2)	64 (4)	0.27
SSA Ro52	3 (3)	12 (0.8)	0.03
SSA Ro60	3 (3)	12 (0.8)	0.03
SSB	3 (3)	15 (1)	0.06
Total number of positive ANA sub-specificities	16 (16)	148 (10)	0.0047

aPL: antiphospholipid antibodies; CRP: C-reactive protein; DCCT: Diabetes Control and Complications Trial standardisation of HbA1c; DVT: deep venous thrombosis; IFCC: International Federation of Clinical Chemistry standardisation of HbA1c; IgA: immunoglobulin A; IgG: immunoglobulin G; IgM: immunoglobulin M; IGT: Impaired Glucose Tolerance; NA: not applicable; OGTT: Oral Glucose Tolerance Test; RNP: ribonucleoprotein; Sm: Smith antigen; SSA: Sjögren antigen A; SSB: Sjögren antigen B.

Data are presented as mean ± SD, median [interquartile range] or number (%). If not otherwise stated patient data were retrieved at the follow-up visit.

### Logistic regression analyses

The associations (odds ratio [OR] and 95% confidence interval [CI]) of HbA1c, fasting glucose, 2-h glucose and aPL IgG positivity were 1.04 (1.03–1.05), 1.14 (1.02–1.28) and 1.13 (1.04–1.22), respectively. These associations remained significant after adjustments for age, gender and smoking habits [1.04 (1.02–1.06), 1.14 (1.00–1.27) and 1.12 (1.04–1.21)], respectively.

## Discussion

The main finding in this post hoc analysis of the PAROKRANK cohort is that dysglycaemia is significantly more common in subjects with aPL IgG positivity. Furthermore, previously undetected diabetes identified by OGTT was the main contributor to this difference. Importantly, there were significant associations between glucose levels, expressed as HbA1c, fasting glucose and 2-h glucose, and aPL IgG. These associations were evident, especially for post-load glucose levels in subjects with previously unknown dysglycaemia, even following adjustment for age, gender and smoking, confounders known to be related to either diabetes or aPL positivity.^22–24^

To the best of our knowledge, only few studies have reported on an association between aPL and dysglycaemic conditions. In 1989, Hendra et al. measured the frequency and titre of aCL antibodies in patients with diabetes (type not specified) with and without CVD and in 2500 healthy controls.^12^ They concluded that although there is an increased frequency of low IgG and IgM aCL titres in patients with diabetes, macrovascular disease was not associated with these titres.^12^ Subsequent studies mainly reported on higher aCL IgG levels in small populations of type 1 diabetes, without any clear association with vascular complications, while other reports on diverse aPL groups including both type 1 and type 2 diabetes revealed conflicting results regarding their association with macrovascular disease.^13–17^ Two studies reported a positive correlation between aPL and neuropathy due to diabetes.^25,26^ An etiologic role was suggested since they appeared to be correlated to the extent and severity of nerve destruction.^25,26^ These results were summarised in a review reporting on endocrinological manifestation of APS, including diabetes, which highlighted the contradictory results of available data.^13^ Among these studies, dysglycaemia was characterised by means of OGTT only in one report with results resembling the present.^27^ Thus, data from previous studies are contradictory and affected by various drawbacks.^18^ First, some of these studies were carried out in limited sized populations. Second, a wide variety of aPL were measured, and in most of the reports the actual titres in patients with diabetes were low or moderate. Third, the analytical methods for aCL were not standardised according to the recent international consensus statement.^10^ Finally, the mix of type 1 and type 2 diabetes in the majority of these reports may be misleading since the two conditions have major different pathogenesis.^13^

Some of these limitations are overcome in the present report. First, both aCL and aβ_2_GPI were assessed, and positivity for either of the two is referred to as ‘aPL positivity’, since they are considered ‘criteria’ antibodies for APS. Measurements were carried out by means of a standardised method, measuring several isotypes, and IgG positivity, but neither IgM nor IgA was more common in dysglycaemia. This is in line with previous reports that aPL of the IgG isotype seems more associated to the pathogenicity of occlusive vascular events than IgM and IgA.^28–31^ Furthermore, type 1 diabetes was excluded and only IGT and type 2 diabetes were included, in order to selectively accounting for alteration possibly related for a atherothrombotic phenotype associated to insulin resistance.^6^

In the present study, aPL IgG positivity was investigated in relation to a well-known prothrombotic condition, dysglycaemia, to encourage a comprehensive approach to the overall thrombotic risk of these subjects.^11^ Since the prothrombotic alteration has been suggested to increase in line with the insulin resistance, the present findings might mirror this continuity, indeed showing an association between aPL IgG and dysglycaemia, a majority previously undetected.^6^

Moreover, this cohort included patients with a first-time myocardial infarction and matched controls. First myocardial infarction was more frequent in aPL IgG positive subjects, in line with reports which showed a strong association between aPL positivity and a first myocardial infarction.^20,32^

The correlation between aPL IgG positivity and dysglycaemia raises important issues regarding treatment strategies. Either anticoagulation at a target international normalised ratio (INR) 2.0–3.0 or high intensity of 3.0–4.0 or combined therapy with low-dose aspirin (LDA) plus anticoagulation at a target INR of 2.0–3.0 are recommended for secondary prevention of arterial events in APS.^11^ However, it is not clear which of these three strategies is better, and the risk of bleeding and other thrombotic risk factors should be taken into consideration. Data from a recent retrospective report from the New York Presbyterian Hospital and APS ACTION cohort suggested that dual therapy with LDA plus anticoagulation at an INR of 2.0–3.0 decreased the rate of recurrent arterial events when compared with anticoagulant and antiplatelet therapy alone (6.9% vs 23.7% and 37.2%, respectively).^33^ However, this specific point remains quite controversial, and data are even more limited on coronary artery thrombosis specifically.^32,34,35^

There are several strengths with this report. First of all, it presents data from a large, representative, multicentre study, in which participants were carefully characterised with recommended analytical tools and the patient–control matching might help eliminate potential confounders. All three isotypes of aCL and of aβ_2_GPI (IgG, IgM and IgA) were measured by a standardised method and cut-offs for ‘positivity’ were in agreement with the most recent APS classification criteria.^10,36^ Moreover, characterisation of the glycaemic state was extensive and based on a standardised OGTT. Recent analyses of the European Survey of Cardiovascular Disease Prevention and Diabetes (EUROASPIRE) cohort, including approximately 4000 patients, reported that this test is the most sensitive and predictive test for the diagnosis of dysglycaemia in patients with coronary artery disease.^37^ Importantly, 2-h post-load glucose is the only test that discloses IGT, and it seems to identify a different type 2 diabetes patient than fasting and HbA1c.^38,39^ In this study, one quarter of participants, mainly first-time MI patients, had previously undetected dysglycaemia, confirming that it is an underdiagnosed and high-risk condition.^40^

There are some important limitations as well. First of all, the frequency of all aPL detected is low (6% of the whole population), although in line with the prevalence in the general population, ranging from 1% to 5%.^41^ Due to the low numbers, we did not further evaluate the aPL-dysglycaemia association in MI-cases and controls separately. An additional limitation is the lack of the functional lupus anticoagulant test, since citrated plasma was not collected. Moreover, the current report measured aPL in a single determination. Thus, we cannot exclude that the observed antibodies are transient, even though aPL have been shown to remain stable over time in approximately three quarters of cases, and there is to date no scientific evidence that transient aPL are not a risk factor for thrombogenesis during the period that they circulate.^41–43^ In fact, aPL positivity has been suggested to predict both arterial and venous vascular events in prospective cohorts, independently from other thrombotic risk factors.^44^ Finally, the observational nature of this study prevents from drawing conclusions on causality.

In conclusion, the present study indicates that a subgroup of patients with dysglycaemia are carriers of a previously neglected prothrombotic risk factor, due to the presence of IgG aPL. Subsequent subgrouping of dysglycaemic patients by aPL IgG testing could identify patients at increased risk of thrombosis. Further studies are needed to verify the present findings and to investigate if antithrombotic therapy may reduce the risk of vascular complications in aPL positive subjects with dysglycaemia.
